# New Jersey State Dental Society

**Published:** 1890-12

**Authors:** 


### NEW JERSEY STATE DENTAL SOCIETY.—TWENTIETH ANNUAL SESSION.

THE Twentieth Annual Meeting of the New Jersey State Dental Society was held at the Coleman House, Asbury Park, N. J., on Wednesday, Thursday, and Friday, July 16, 17, and 18, 1890.

*Wednesday, July 16.—Morning Session.*

The president, S. C. G. Watkins, D.D.S., in the chair.

The meeting was opened with prayer by Rev. Dr. Bomberger, of York, Pennsylvania.

The president, Dr. S. C. G. Watkins, then called to the chair Vice-President Adams, and read his annual address, as follows:

PRESIDENT'S ADDRESS.

Gentlemen and Friends of the New Jersey State Dental Society,—It becomes my pleasure and duty to preside over this body, which to-day reassembles in convention, celebrating its twentieth birthday. In former years we have heard so much about old historic ocean that it seems needless for me to-day to repeat anything about her turbulent bosom or her wild waves, which many of you, no doubt, will try to conquer before you leave her shore. But, gentlemen, we have great reason to rejoice on this occasion, and to thank God for his goodness in permitting us to meet here on this our twentieth anniversary with unbroken ranks. Every association, in fact every individual, has a mission to perform. Our special work is the alleviation of human suffering. What greater incentive could we have to stimulate the power within us to action? Surely it is the grandest work in which the skill of man can be enlisted. We are laborers in the field of human progress, and it is only through an organized association like this that dental thought can be thoroughly and usefully applied. The advantages of personal interchange of ideas, which arise by the discussions in our assembles, are not attained by merely reading the journals in the seclusion of our offices. There is something in the friction of mind against mind which stimulates to action, induces investigation, and leads to higher attainments. Much has been accomplished, yet we are only in our infancy. We must grow.

There is a demand for advancement, for a higher qualification, a broader curriculum, and an extension of time in our colleges. It is a problem whether the increased number of colleges is an advantage to the profession or not. I cannot but view the increase of schools with alarm. Are they created because of necessity, or through jealousy and personal preferments? Although the number of colleges has augmented, the Association of Faculties has recognized the demand for a higher standard in dental education, and has increased the length of the term to five months for three successive years, which is a step in the right direction. I would recommend to this Society that we put ourselves on record as favoring this term of pupilage, with a lecture course of not less than seven months in each year, and we should insist upon an outside Board of Examiners. For it is at the college-door that we should guard the sacred precincts of professional life. Instead we have had to place this matter in the hands of our State Examining Board, and to ask it to draw the line, so far as professional honor in this State is concerned, by examining all who desire to practise dentistry within our borders, even though they be graduates. Also that the Examining Board have the power conferred on it by the laws of the State to say which college shall be deemed reputable.

At the semi-annual meeting, held at Montclair, January 11, the Committee on Legislation was instructed to procure the passage of an amendment to our State law regulating the practice of dentistry, which was successfully done, and I now have the pleasure of congratulating you upon your law as being second to none. These laws clearly define the requisites for a dental practitioner, and they protect the interest both of the practitioner and the public. I would suggest that a vote of thanks be tendered the legislative committee for its efforts, which required a great deal of sacrifice. I will not say more in regard to legislation, as I am fully aware that the committee is anxious to give you the results of its labors. I would invite your attention to the well-known fact that the American Dental Association will meet at Excelsior Springs in August. Also that the International Medical Congress, with its dental section, will meet in Berlin. I hope that our Society will be well represented in both of those conventions.

It is a pleasant privilege for me, as president of this Society, to welcome so many eminent members of our profession from sister States who have come to mingle with us, and give us the benefit of their investigations on this occasion. Gentlemen, we appreciate the sacrifices you have made, and we cordially invite you to participate in our discussions with perfect freedom. I am proud to be able to announce to you to-day that there is neither strife, jealousy, nor contention within our ranks; that it has been said that we were an overgrown mutual admiration society, and express the hope that this harmonious condition of affairs will continue to exist for the future good of the organization. I now thank you, gentlemen, for the honor you have conferred upon me, and I now assume the duties of the office of president with some apprehensions. I hope I may receive your hearty co-operation and support.

On motion, the president's address was referred to the following committee: Drs. B. F. Luckey, Fred. A. Levy, and Albert Westlake.

Dr. J. A. Osman then read an essay entitled "From Another Stand-Point." (For Dr. Osman's paper see page 739.)

*Dr. L. Ashley Faught.*—I have listened with the deepest interest to the reading of this admirable paper by Dr. Osman. The theme he has chosen and the time of its presentation seem to me to be most opportune. The subject of dental education has earnestly engaged the attention of the profession at different periods during the last twenty years. Its claim for consideration has, however, been most persistently presented at dates which correspond with a decided change in the stand-point of observation; and each new outlook seems to have been taken at the beginning of a new decade. Even the elements of crude dentistry began to crystallize out of a mere apprenticeship into a higher organization about the year 1840; and the marked extensions of the collegiate system then established tallies with the years 1850 and 1860.

Half a century of experience stands on the records, and what is written and said at the opening of this decade, if it follow the rule, will bring us to another stand-point in the year 1900. We have been striving for perfection. Can we feel the present step to be final? Can we, looking backward, outline to-day that which will stand in the future? These are pregnant queries, for it would seem from the stand-point of the essayist that our further progress is to be but cyclical. Our first history is an era in which everything was mechanical. "It was the day of the reign of 'ingenuity,' when the man who was 'handy' with tools was necessarily a 'born dentist,' and took to it as a mechanical operation." In the course of human events we passed from the mechanical to the mental. Scientific instruction was considered necessary to be able to practise, to procure dignity, respect, and recognition. That the latter step should have pushed almost all evidences of the former to the wall, even to the point that the highest (?) *dental* attainment is to become a graduate in medicine, is not a matter of surprise. We are by the paper just read now invited to return from the error of our way, and again develop the mechanical; to pay less marked attention to theory; to give the attention to the practical which it should receive, and to make our dentists hand-wise rather than head-wise.

Such reversion is, even as the essayist suggests, because "no two people see the same thing in exactly the same light;" but strange it is "how *very* differently different people see the same thing."

Our fathers did not regard the mechanical as the most valuable training, but we view it as a thing very much to be desired. Difference must be in cultivated perception, and cultivated perception is the outgrowth of scientific training. I cannot, therefore, entirely agree with the sentiments of the essayist which I have just quoted, the tenor of which is to train the dental recruits more and teach them less; for I conceive that our highest good lies not in developing either the mental or the mechanical at the expense of the other, but in a wise and judicious blending of the two. While it is true that ours is a surgico-artistic profession, and that mechanical operation constitutes the largest part of our duties; that we are engaged more with the mechanical than with the medical, more with the prosthetic than with the therapeutic; yet the coming dentist will practise as an adviser as well as an operator, and must be a scientific man. What he should seek to do is to blend the scientific with the practical; but this practical education cannot be taught, it can only be acquired. It is therefore a fair conclusion "that the objections of the utilitarian to the higher scientific education hold good in only one respect,—*i.e.*, in the deficiency of practical training, and the proper union of science and work. This deficiency under the present system is indefensible, and must be improved. But we must defend it against his further attacks, because scientific education is valuable, as, first, including nothing irrelevant or unnecessary; second, as being required for mental discipline; and, third, as tending to elevate the morals of dental education." How this blending is to be best accomplished I am sure that those engaged in the active work of teaching can more properly suggest, and I have all faith and confidence that it will receive at their hands all merited consideration.

There are, however, concomitant evils connected with dental education which, from our stand-point outside the ranks of teachers, demand indication. I know it is easier to pull down than to build up, to criticise than to create; and yet the true and permanent elevation of our profession requires that we consider not men and institutions, but that we speak the truth in love.

We want, and must have, at the present time, less rivalry between our institutions of learning. The effort should not be for popularity, or to rank as the best; but the aim should be the same in all,—to help one another preserve the honors of the degree, and to properly prepare material for the dental ranks. The list of matriculates will bear large pruning, for many are admitted who show a most unhealthy tone at the very start by wishing to know "how to get a degree with the least expenditure of time and money;" and many are graduated whose dental diplomas are, as indicated by another, "simply a lie. It certifies that the purchaser possesses the qualifications necessary to practise dentistry, which his instructors must have known, if they knew anything, that he did not possess." I trust that this will not arouse antagonism in the breast of those who are so nobly devoting their lives to the work of education, for I am fully aware that the flaw lies more remotely. Self-preservation is a law supreme, and to obey it is but natural. Truly, though, we need not so many colleges, and if quite a number could be done away with and the rest relocated with non-conflicting territory, and these heavily endowed, a great step would be taken towards the eradication of corruption and the establishment of such institutions as need only receive for preparation and graduate those fitted to practise dentistry. I desire to lay especial stress upon this need of endowment. Money has been left, by those possessing it, to legal, theological, medical, scientific, and art schools, but not one dollar yet, so far as I am aware, has been given to any dental institution.

I believe, too, that a more healthy tone could be imparted to dental colleges if their boards of trustees were composed of their own local alumni. These gentlemen, having no interest in the graduation of large classes and a decided interest in suppressing incompetency, would, by their consideration of the names recommended by a faculty for graduation, never allow such recommendation to be equivalent to the issuing of the mandamus for conferring the degree. Operating in somewhat similar manner, much can be expected from the work of those State boards of registration and examination in dentistry which may be in the future created to exist under such State dental laws as was the happy lot of this Society to succeed in having passed in April of this year.

I cannot let this opportunity pass without also calling attention to a needed reform in dental education which, to my mind, has a most *practical* bearing. In reviewing the issued yearly announcements of the colleges, and the public advertisements of them in the dental journals, the eye is arrested by the uniform tendency to have long lists of demonstrators. This, at first glance, is assurance that the students are most carefully taught in the manipulative department. Careful analysis shows the fallaciousness of such promised instruction, for many of these demonstrators are, as a rule, recent graduates, and who for the most part have not that mature experience which here, more than in any other instructor, should be possessed by him who assumes to guide the inexperienced. Such long lists should be done away with and replaced by one or two individuals who, from long experience in actual practice, rank coequal with the gentlemen holding the professorial chairs. The teaching in this department is vital in importance, and should never be intrusted to the novice.

The clinical teaching in dental education is the stronghold of the practical, and should not be reduced, as it too often at present is, to a mere exhibition of the introduction of finely-finished gold fillings before students by noted practitioners. It is through seeing others perform varied operations that suggestion is had of those little essentials which, to my mind, cannot be obtained in any other way. I would, therefore, have the clinic list and day of each college made not as full as possible, but *full*, and special effort exerted to so maintain it.

I am heartily in accord with the second reform mentioned in the paper,—that we educate the masses, the busy people; because I believe that all education in dental matters tends to help attain for ourselves, or for our successors, the position we seek; and yet I cannot but feel that the knowledge possessed by the public is much more advanced than it is willing to admit, for we have talked and taught these things for many years. The real difficulty in the attitude of the public is, that dentistry is without legal standing; and the public seek and accept a service that is neither intelligent, scientific, successful, or safe, so largely proffered them by what are understood as "legally-qualified dentists." A premium is thus placed upon such poor dentistry, which, in the present legal condition of the profession, will not be removed, and colleges are encouraged to produce men able to measure to this standard, for all efforts to produce better men will be futile until a change is accomplished in the legal standing of the calling, and an appreciation shown by the public in the fees they would be willing to render for their services. Men are not easily found willing to reach a higher point than that at which they will be remunerated by the public, especially when their diplomas, though reading "we do most freely and fully grant and *make sure* all the peculiar rights, honors, and privileges pertaining to said degree," *do no such thing*. That all this should be so is passing strange, and yet it is true; for in our educated and educating age, it is almost impossible to believe that ignorance has anything to do with the reproach that men do practise dentistry illegally who are thoroughly unqualified, as well as do many legally who are comparatively incompetent. The cause of the existence of these evils lies in the non-application of the knowledge possessed by the public, and the consequent willing acceptance of such service; and they will continue until the people lift their demands to a level with their knowledge. It has been advanced and believed that the growing intelligence of the general public would eventually reform matters, and we have consoled ourselves by directing our efforts to their instruction in subjects pertaining to dentistry. Much good has been done in this way, but the startling truth is that the end for which the instruction was given is far from being attained. As the public will not use this knowledge, and select and properly remunerate worthy practitioners, it is a fact to be recognized that the practice of dentistry will only be raised to a safe standard, and mountebanks and quacks driven from their deceptive practices, by an invocation of the strong arm of the law. We need a universal recall of all State laws that are full of faulty enactments, and corrected ones with more extensive powers passed. Such concerted action, coupled thereafter with prompt prosecution of all violators, will do more for living, concrete dentistry, in the minds of the public, than all the most scientific teaching, in the most guarded and attractive language, in the public press. That this is true is manifested in the fact that even the most ignorant receive the services of a physician with reverence, respect, deference, and with willingness to remunerate, if possible, simply because it has dignity through legal standing.

We therefore need not to look so much at the condition of the public as we do to consider ourselves, to organize more fully on a broad, learned, professional foundation; to establish a conservative love for the general interests of the profession, and a fraternal love for each individual brother that will jealously protect his interests as our own.

*Dr. R. McL. Sanger.*—The attention which the leading minds of the profession are now giving to the subject of higher dental education is one of the best indications possible of the advance of dentistry. The papers that have been recently read before different dental societies, and have received a hearty welcome from them, notably the paper read by Dr. Kirk, of Philadelphia, before the First District Dental Society of New York, together with the able discussion of the same, have made us feel that great things might be reasonably expected of the dentist of the future.

But Dr. Osman, in the very able paper to which we have been listening, has directed our attention to an entirely different view of the subject of dental education,—to wit, the education of the public. The thoughts presented are well worthy of careful consideration, and the results that would follow a practical application of the views of the writer make a beautiful picture in the imagination. If we have been led to take a rosy view of the coming dentist, Dr. Osman now supplements it by a picture of an educated public in a setting of equally roseate hues. We are charmed with the prospect of having patients in our chairs who know so well what ought to be done that they are perfectly willing to leave the management of their individual cases in our hands.

It would be impossible in the short time allowed for discussion to review the whole subject as it has been presented to us, and I will therefore speak of one or two points which have not been definitely mentioned, yet are suggested by the paper, and are in accord with its general purport. There is truth in the saying that a little knowledge is dangerous. You will all agree with me in the statement that a patient who knows just enough to think he knows more than he really does is an unmitigated nuisance. Yet, when we have said this, we must still allow that there are some things in reference to the care of the teeth that should be more widely known than they are.

Very much can be accomplished in this direction on the lines suggested by Dr. Osman, but it is in connection with the children that we can accomplish the best results. It is a well-worn statement that, given the control of the children, the future of a generation can be determined, and what has actually been done through instruction in youth is almost incredible. Probably the most remarkable illustration of this is in the training of Hindoo boys. A European of intelligence and education may spend years in India investigating the marvellous caste system of the Hindoos, and be compelled to own at last that he has not fully mastered the details. But the young Hindoo at thirteen years of age has been so thoroughly trained and instructed in the intricacies of the system that, in practice, he has never been known to fail, through ignorance or carelessness, in keeping the minutest points.

Instruction in dental hygiene can be given in the home by parents, of course, and this is being done more and more. But in line with the suggestion that the State Dental Society should take up the work of sending out information through the press, I would suggest that something could be done to advantage by introducing instruction in dental hygiene into schools, either by encouraging short talks to the scholars at intervals, or having the information we wish to give put into the form of lessons. This is being done in matters of general hygiene, and physiology is now commonly taught, so that the way is already prepared for the introduction of this teaching.

The other point which I will mention is one of some delicacy, because it concerns our medical brethren. It must be said in all kindness that there is need of more exact dental knowledge on the part of the family physician. As it is now, the dentist is often put to great inconvenience in doing what is best for the patient by the faulty advice given at home by the medical attendant. It frequently happens that patients come into a dental office to have a number of teeth extracted, and wish to take an anæsthetic to escape the pain which will otherwise be unavoidable. In these cases there is usually a large proportion of roots without crowns, and the dentist agrees that an anæsthetic is desirable, not only because pain will be avoided, but also because the shock to the nervous centres will be greatly lessened, much to the benefit of the patient. But when he makes his arrangements for giving the nitrous oxide, the patient disconcerts him by saying that she cannot take it; her physician told her she might take ether or chloroform, but not gas. Here is a quandary. In almost every case the gas can be taken without the least inconvenience, while the danger of giving either of the other anæsthetics for such an operation would make it inexcusable, and the shock from operating without an anæsthetic would be considerable. The physician may never have seen gas given, or at least infrequently, while the dentist has daily experience in its use. Here is an awkward position. The operator does not wish to give ether or chloroform, but to give gas. To express his opinion brings him into conflict with the medical attendant, and still the patient calls for some anæsthetic.

Another point in which the dentist is hampered by the advice of the physician to the patient, and one involving oftentimes more serious consequences, is in connection with ulcerated teeth. A patient comes into the office with a face swollen from accumulation of pus and the accompanying inflammation, the source of the trouble being a diseased tooth. The indication seems plain to give an outlet to the pus by extraction, the tooth being past saving. The advice to have this done without delay is given, but it is met by the statement that the family physician has given orders that the tooth must not be drawn until the swelling has subsided. The patient returns to the physician to have the head incased in a flaxseed poultice, followed in a day or two by a plunge of the bistoury, usually from the outside. The result, as we all know from experience, is frequently seen in lazy abscesses, which continue to discharge until the offending tooth is finally removed, leaving ridges of cicatricial tissue as a life-long reminder of a serious mistake somewhere.

But it is not necessary to multiply examples; we meet with them daily in our practice. The question is, how can we remedy this? Might not the State and local societies do a little good missionary work right here by inviting the physicians to take part in our meeting and discussions, or by exchanging papers with the medical societies in our midst? Such a course would not only make life more tolerable for the dental practitioner, but would elevate him in the respect and esteem of his medical co-laborers.

(To be continued.)

